# Immune-related gene signature associates with immune landscape and predicts prognosis accurately in patients with Wilms tumour

**DOI:** 10.3389/fimmu.2022.920666

**Published:** 2022-09-12

**Authors:** Xiao-Mao Tian, Bin Xiang, Li-Ming Jin, Tao Mi, Jin-Kui Wang, Chenghao Zhanghuang, Zhao-Xia Zhang, Mei-Ling Chen, Qin-Lin Shi, Feng Liu, Tao Lin, Guang-Hui Wei

**Affiliations:** ^1^ Department of Urology, Children’s Hospital of Chongqing Medical University, Chongqing, China; ^2^ Ministry of Education Key Laboratory of Child Development and Disorders, Chongqing Key Laboratory of Pediatrics, National Clinical Research Center for Child Health and Disorders, China International Science and Technology Cooperation Base of Child Development and Critical Disorders, Children’s Hospital of Chongqing Medical University, Chongqing, China; ^3^ Chongqing Key Laboratory of Children Urogenital Development and Tissue Engineering, Chongqing, China

**Keywords:** Wilms tumour, immune signature, tumour immune microenvironment, prognosis, immunotherapy

## Abstract

Wilms tumour (WT) is the most common kidney malignancy in children. Chemoresistance is the leading cause of tumour recurrence and poses a substantial therapeutic challenge. Increasing evidence has underscored the role of the tumour immune microenvironment (TIM) in cancers and the potential for immunotherapy to improve prognosis. There remain no reliable molecular markers for reflecting the immune landscape and predicting patient survival in WT. Here, we examine differences in gene expression by high-throughput RNA sequencing, focused on differentially expressed immune-related genes (IRGs) based on the ImmPort database. *Via* univariate Cox regression analysis and Lasso-penalized Cox regression analysis, IRGs were screened out to establish an immune signature. Kaplan-Meier curves, time-related ROC analysis, univariate and multivariate Cox regression studies, and nomograms were used to evaluate the accuracy and prognostic significance of this signature. Furthermore, we found that the immune signature could reflect the immune status and the immune cell infiltration character played in the tumour microenvironment (TME) and showed significant association with immune checkpoint molecules, suggesting that the poor outcome may be partially explained by its immunosuppressive TME. Remarkably, TIDE, a computational method to model tumour immune evasion mechanisms, showed that this signature holds great potential for predicting immunotherapy responses in the TARGET-wt cohort. To decipher the underlying mechanism, GSEA was applied to explore enriched pathways and biological processes associated with immunophenotyping and Connectivity map (CMap) along with DeSigN analysis for drug exploration. Finally, four candidate immune genes were selected, and their expression levels in WT cell lines were monitored *via* qRT-PCR. Meanwhile, we validated the function of a critical gene, NRP2. Taken together, we established a novel immune signature that may serve as an effective prognostic signature and predictive biomarker for immunotherapy response in WT patients. This study may give light on therapeutic strategies for WT patients from an immunological viewpoint.

## Introduction

Wilms tumour, also named nephroblastoma, is the most prevalent kidney tumour, accounting for more than 90% of all kidney tumours in children (1). With the advancement of multi-modal therapy including surgery, chemotherapy, and radiotherapy in past years, the overall survival rate of children with Wilms tumour has increased to almost 90% (2–4). The 5-year survival of patients with stage I-III was close to 90%, while that with stage IV was less than 80% (5). Wilms tumour has a 15% recurrence probability, and the long-term survival rate of recurrent tumour is about 30% to 50%, according to studies (6, 7). In addition, treatment-related issues continue to be a challenge for WT patients (8–10). WT patients suffered a dual burden in balancing treatment advantages and adverse effects (11). It’s tough to make significant improvements merely by improving the current therapies (multi-modal therapy). Thus, immunotherapy and targeted therapy, both of which have excellent precision and few complications, have started to be explored for WT treatment (12, 13). Despite the fact that immunotherapy and targeted therapy are rarely applied in clinical practice, several clinical trials are now underway around the world (https://clinicaltrials.gov/).

It should be noted that individuals who share the same clinical phenotype and receive the same treatments could have a distinct prognosis, suggesting that WT is far more complex and touches many more aspects of biology than was previously appreciated. Individuals’ diverse responses to cancer therapy have been largely attributable to tumour cell genetic differences (14). Traditional histological categorization schemes failed to accomplish the primary goal of directing clinical management and stratifying patients outcome, especially in individuals without any unfavorable factors (15). Thus, novel biological markers that can improve risk categorization for WT patients are desperately needed in order to guide more personalized treatment decisions. Emerging evidence suggests that cancer cells could regulate immune cell activities and cytokine release, affecting anti-tumour immunity and immunological evasion (16). In addition to the fundamental characteristic of the tumour cell themselves, the TME is critical for tumour progression, and immune cells in the tumour niche play a critical role in tumourigenesis, therapeutic response, and immunological evasion (17). A systematic investigation of immune phenotypes in WT microenvironment is a promising approach for a greater understanding of anti-tumour immune responses and to guide the development of effective immunotherapy. Although individual genes as biomarkers may be of limited value, polygenic riskscore has shown that prediction is sufficiently accurate for several applications (18, 19). Assisted by high-throughput sequencing technology and bioinformatics, more accurate and reliable prognostic signatures could be developed for specific patients (20–24). However, no relative study has been carried out to predict and guide the response to immunotherapies for WT patients. Therefore, the development of an immune biomarker, which reflects the immune status and achieves great predictive power toward WT patients’ outcomes upon a complete list of IRGs is warranted. Such a targeted study has the potential to improve clinical outcomes.

**Figure 1:**
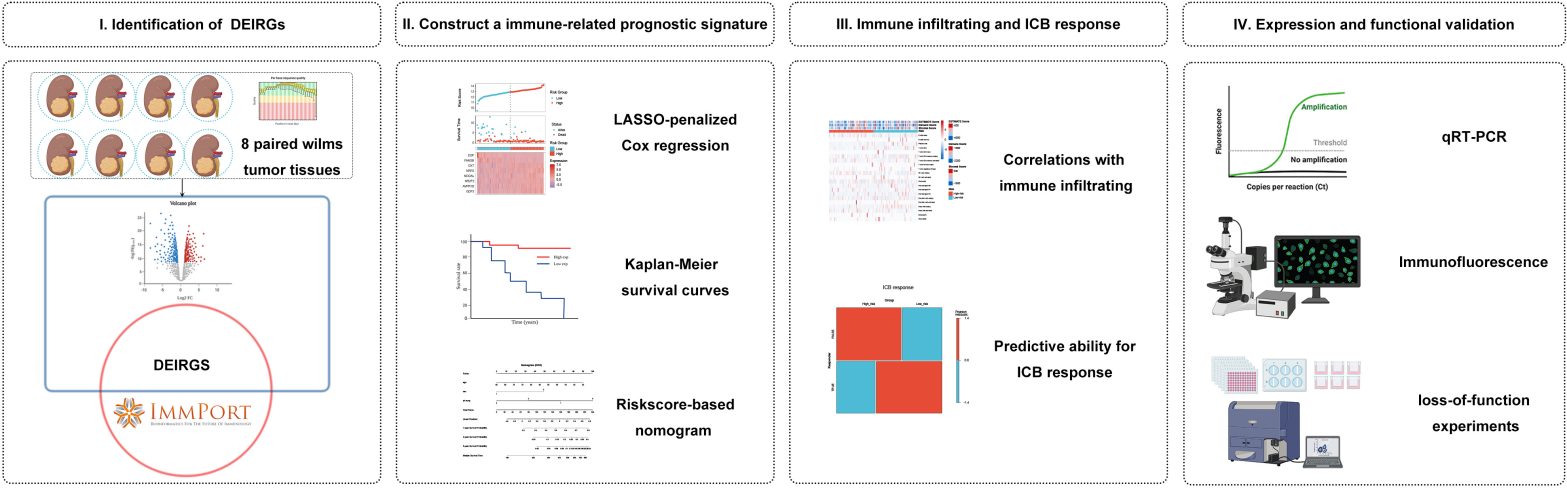
Analysis flowchart. **(I)** Identification of differentially expressed immune-related genes (IRGs) by tumour tissue sequencing in Wilms tumour and the ImmPort database. **(II)** Construction and validation of immune-related gene signature in Wilms tumour. **(III)** Association with immune infiltration and predictive ability for the response to immunotherapy. **(IV)** Expression validation and functional validation.

We hypothesized that immune-related gene signature could serve as a valuable prognosis biomarker and allow precise TIM characterization in WT patients. Here, we first identified differentially expressed IRGs based on a complete list of IRGs and validated them by high-throughput RNA sequencing. Then, based on the TARGET cohort, the immune signature associated with patient survival and a nomogram combined with immune signature and prognostic clinical risk factors were established. In addition, we used the xCELL, ESTIMATE, and ssGSEA algorithms to evaluate the association between TME immune infiltration and immune riskscore. Lastly, we evaluated the effect of the immune signature for its ability to predict the response to immune checkpoint blockade (ICB) therapies. Overall, the polygenic immune signature has high prognostic clinical utility and effectively predicts immunotherapy response. Thus, our findings can help identify patients who are potentially benefited from immunotherapy and facilitate clinical practice. **Figure 1** shows a schematic representation of the major steps to portray our study more clearer.

## Materials and methods

### Identification of differentially expressed IRGs

Eight paired tumour tissue and adjacent normal tissue were generated from the primary locations of patients with WT for high-throughput RNA sequencing investigation. The GEO (gene expression omnibus) database has been used to store RNA-seq data (https://www.ncbi.nlm.nih.gov/geo/, accession nos. GSE197047). All WT tissues were generated from the Children’s Hospital of Chongqing Medical University. All patients are referred to the COG protocol for diagnosis and treatment (25). The study was approved by the local research ethics council, and all patients or guardians gave informed consent (Ethical batch number: 2022-50). To measure the expression levels of individual genes in mRNA expression analysis, we utilized fragments per kilobase per million mapped reads (FPKM), which was estimated as follows:




$$\text{FPKM}=\frac{\text{total exon fragments}}{\text{mapped reads }\left(\text{millions}\right)\times \text{exon length }\left(\text{kb}\right)}$$

The fragments inside each gene were counted using Stringtie software (26). The edgeR software was then used to produce FPKM values and differential gene expression analysis (27). Finally, to identify differentially expressed genes (DEGs), both |logFC| > 2 and Q value< 0.05 were employed as screening criteria. The ImmPort database was used to get a complete list of immune-related genes (IRGs), which included 1793 genes in total (Details are shown in **Supplementary File 1**) (28). Using this database as a background reference, we obtained a list of differentially expressed IRGs and their associated category (29).

### Gene ontology, Kyoto encyclopedia of genes and genomes, and gene set enrichment analysis

To investigate the potential biological processes of the DEGs from the RNA sequencing results, GO and KEGG pathway enrichment analysis was performed by the R package clusterProfiler (30). GSEA is a method for determining if a collection of genes defined in advance displays statistically significant, concordant differences between two biological states (31). The gene signature’s potential pathways and biological processes between high- and low-risk subgroups were analyzed using GSEA based on Molecular Signatures Database v7.4. The enriched functional GO terms and KEGG pathways were analyzed using C5 curated gene sets. All GSEA analysis was performed using default parameters.

### Identification of survival-related IRGs and construction of an immune-related gene signature

122 patients containing RNA sequencing data and corresponding clinical information were downloaded from the TARGET database (https://ocg.cancer.gov/programs/target), that were recruited to a training cohort (Details are shown in **Supplementary File 2**). For the initial screening of prognosis-related immune genes, univariate Cox regression analysis and Kaplan-Meier survival analysis were performed. We focused on prognosis-related IRGs to construct a prognostic immune gene signature. For the IRGs with prognostic ability, the Cox proportional hazards model with a Lasso penalty (iteration = 10) was employed to find the best gene model utilizing the R package ‘glmnet’ (32). The immunological signature was created by combining the selected gene expression levels in a linear fashion and weighting them according to the Lasso-Cox regression coefficients. The following formula could be used to represent the developed prognostic model succinctly: Riskscore = ∑in(Coefi × Xi). In the multivariate Cox regression model, the Coef reflects the coefficient of relative prognostic IRGs, and the X represents the expression level of each IRG. The formula was applied to calculate each patient’s riskscore, and a cut-off number between high- and low-risk subgroups was determined using the median riskscore. Moreover, Kaplan-Meier survival analysis and time-dependent receiver operating characteristic (ROC) curves were applied to measure the prediction accuracy of this signature.

### Validation of the immune signature

To verify the predictive accuracy of the immune-related gene signature, we used survival analyses and independent prognostic analyses to see if there was a distinction between the high- and low-risk subgroups. Specifically, we retained only patients with full clinical information, including sex, age, race, clinical-stage, pathological type, and progression-free survival (RFS). The distribution of clinicopathological features was also assessed using the chi-square test and visualized using heatmaps in high- and low-risk subgroups. To validate whether the predictive ability of immune signature was independent of conventional clinical characteristics, stratified analyses, univariate Cox regression, and multivariate Cox regression were applied. Subsequently, a nomogram was built using the abovementioned variables *via* the Cox proportional hazards model. Furthermore, the calibration curve and the decision curve analysis (DCA) conducted by the R package ‘rmda’ were applied to assess the accuracy of the nomogram. To verify the clinical value and subsequent immune correlation of this signature, the GSE31403 cohort’s gene expression profiles and associated clinical data were acquired from the GEO database (Details are shown in **Supplementary File 3**).

### Relationship between immune signature and TIM

The Stromalscore, Immunescore, and ESTIMATEscore that reflect the TME-related cell infiltrating degree in tumour tissues of WT were calculated with the R package ‘IOBR’ using the ESTIMATE algorithm (33, 34), which was created on single sample gene set enrichment analyses (ssGSEA). Tumours are heterogeneous tissues in which TME surrounds and interacts with malignant cells, and the TME contains a variety of immunocyte types. To evaluate the heterogeneous cellular landscape of TME, cell types enrichment scores were evaluated. Through ssGSEA, we evaluated the related abundance of immune-cell infiltrations in tumour samples using the Xcell algorithms (35), an R program that performs cell type enrichment analysis from gene expression profiles for 64 types of cells in TME. Then, we compared the difference in infiltrating immune cells in the high- and low-risk subgroups by using the two-sample Wilcoxon test. To discover more about the relation between the signature and TME, Pearson correlation was applied to calculate the relationship between riskscore and immune infiltration calculated by ssGSEA.

### Gene mutation and immune response analysis of immune signature

We investigated the gene signature’s gene mutation landscape and how it affected the potential response to ICB treatment. cBioPortal (http://www.cbioportal.org/) was used to obtain information on copy number changes and mutations. We also analyzed the effects of the mutant genes on the prognosis of WT patients using Kaplan-Meier curves. Immune checkpoint-relevant transcripts including IGLEC15, TIGIT, CD274, HAVCR2, PDCD1, CTLA4, LAG3, and PDCD1LG2 were selected, and their expression levels were retrieved. The correlation between riskscore and immune checkpoint gene expression level was calculated by Pearson correlation. Additionally, the response of tumours to immune checkpoint inhibitors was predicted according to the tumour Immune Dysfunction and Exclusion (TIDE) score, calculated based on a gene expression profile and a computational algorithm (36). In simple terms, the higher the TIDE score, the poorer the treatment effectiveness and prognosis would be.

### Mechanism exploration and candidate small molecule drugs

To continue investigating the possible biological function and pathways between the high- and low-risk subgroups, we performed GSEA analysis to explore significantly enriched signaling pathways. The threshold for statistical significance was set at an absolute value of normalized enrichment score (NES) > 1 and a nominal P-value of less than 0.05. The connectivity map (cMap) database (https://clue.io/) is unravel biology with the world’s largest perturbation-driven gene expression dataset. DeSigN is a web-based bioinformatics tool (https://design-v2.cancerresearch) that uses IC50 data to associate gene signatures with drug response phenotypes. We discovered predicted drugs that may aggravate or avoid the biological processes of tumours according to the up-regulated and down-regulated genes when comparing the high-risk and low-risk subgroups. With an FDR value of less than 0.05 and an enrichment score ranging between -1 and 0, the prospective drugs could be served as a novel target candidate for WT patients. These putative drugs’ 3D structural images were acquired from the PubChem database (https://pubchem.ncbi.nlm.nih.gov/).

### Real-time polymerase chain reaction

Total cellular RNA from three WT cells (Wit-49, WT-CLS1, and SK-NEP-1) and normal renal epithelial cells (293T) were obtained using the Simply P Total RNA Extraction Kit (BioFlux, China) following the manufacturer’s guidelines. The methodology of RT-PCR was performed in accordance with the previous literature (37). The expression of candidate genes (NRP2, EGF, NODAL, and NR2F2) was based on the formula 2^^-ΔΔCt^. **Supplementary File 4** lists the primers used.

### Functional validation of the selected gene

NRP2 was selected for further functional validation. An immunofluorescence assay was conducted as described in previous studies (38). The siRNA targeting the human NRP2 gene and the negative control siRNA were designed and synthesized by Tsingke (Beijing, China). Cell proliferation Assay was tested using the CCK8 Assay (MCE, HY-K0301). Cell migration was studied using the scratch test and Transwell assay (Biozellen, B-P-00002-4, China). Cell cycle phase distribution and cell apoptosis rate were determined by flow cytometry using a BD detection kit.

### Statistical analysis

The experimental results were analyzed using GraphPad Prism Software. R software version 4.0.3 was used for all bioinformatics analyses and R packages. The significance level is indicated by single, double, and triple asterisks, as well as ns (*, **, and *** indicate a significance level of 0.05, 0.01, and 0.001, respectively; and ns indicate no significant level).

## Results

### Identification of 117 differentially expressed IRGs in patients with Wilms tumour

**Figure 2:**
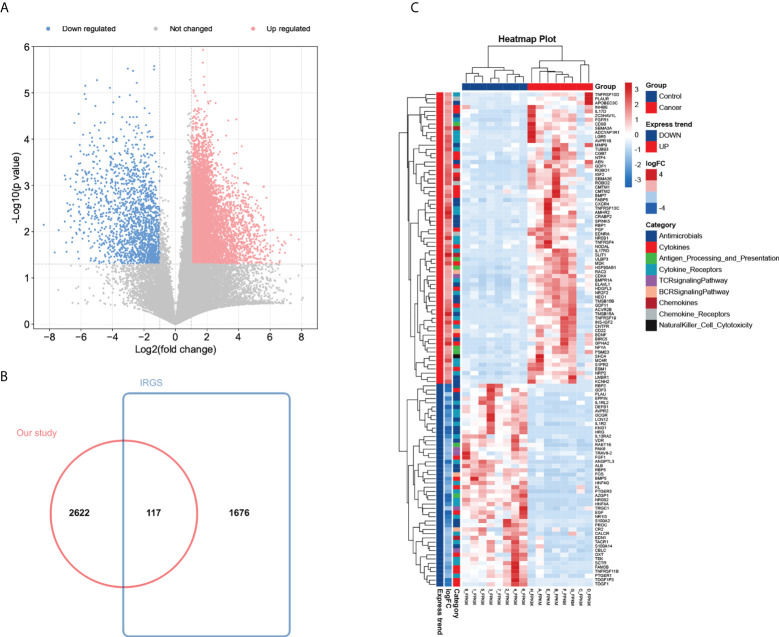
Identification of 117 differentially expressed IRGs in patients with Wilms tumour. **(A)** Volcano plot of differentially expressed mRNAs by tumour tissue sequencing. **(B)** Wayne Figure of mRNA sequencing data versus the ImmPort database. **(C)** Heatmap and clustering analysis of 117 differentially expressed IRGs in patients with Wilms tumour. -4 and 4 represent fold change. High expression is indicated by red, whereas low expression is indicated by green. Different colors represent different immune categories.

To construct an mRNA profile database of WT patients, we collected eight paired clinical specimens, including tumour tissues and adjacent non-tumour tissues to perform high-throughput mRNA sequencing. A volcano plot was used to show DE-mRNAs’ distribution (**Figure 2A**). The expression of DE-mRNAs was shown *via* a heatmap (**Supplementary Figure 1A**), and hierarchical clustering revealed the differential expression pattern between tumour tissues and corresponding non-tumour tissues that distinguished tumour from normal tissue. Most DE-mRNAs had increased expression in WT versus normal samples (2743 DE-mRNAs were identified, including 2009 up-regulated and 734 down-regulated mRNAs in WT).

To investigate the possible biological functions of DE-mRNAs in WT, GO and KEGG pathway enrichment analyses were constructed. The enrichment analysis results are shown in **Supplementary Figures 1B and C**. Many biological processes relevant to malignant phenotypes were considerably enriched, such as the p53 signaling pathway and ‘cell cycle’ pathway (**Supplementary Figures 1D, E)**. These DE-mRNAs were correlated with NK cell and CD8^+^ T activation, according to the GSEA analysis representing cell states and perturbations in the immune system (**Supplementary Figure 1F**). These findings indicate that these DE-mRNAs are linked to tumorigenesis and development, as well as being engaged in the immune response to some level.

To determine the expression differences of IRGs between tumour tissues and adjacent normal tissues, we focused on immunological aberrancies by matching the DEGs set with a list of immune‐associated genes from ImmPort, and ‘jvenn’ diagram was utilized to screen out 117 immune-related DEGs (**Figure 2B**). Heat-map showed the expression level of 117 immune-related DEGs (including 69 up-regulated and 48 down-regulated immune-related DEGs in WT) measured by RNA-seq, which reflect nine immunity-related categories (**Figure 2C**).

### Identification of 12 prognostic IRGs and construction of an immune signature based on the TARGET dataset

**Figure 3:**
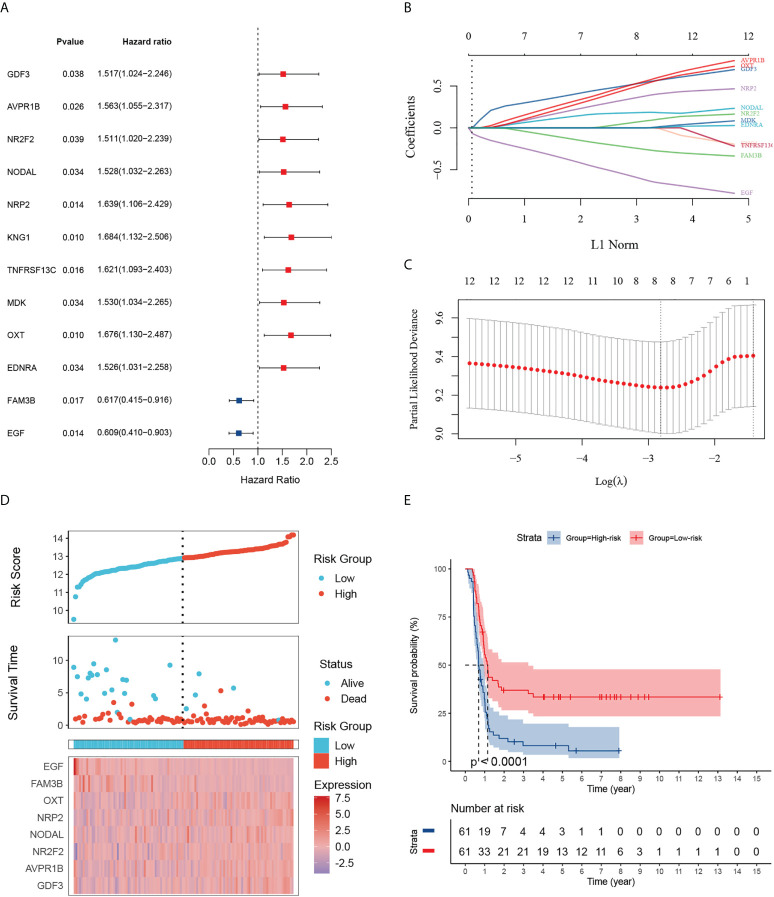
Identification of 12 survival-related IRGs and construction of an immune-related gene signature. **(A)** The forest map shows 12 genes significantly correlated with progression-free survival in the univariable COX regression analyses. **(B)** The trajectory of each independent variable. The log value of the independent lambda is represented on the horizontal axis, while the coefficient of the independent variable is represented on the vertical axis. **(C)** Partial likelihood deviance of variables revealed by the Lasso regression model. The two vertical dotted lines on the left and right, respectively, reflected optimum value according to the minimum and 1-SE criterion. The red dots reflected partial likelihood of deviance values, the gray lines represented standard error (SE). **(D)** Distribution of the riskscore, the associated survival status and the gene expression heatmap of the gene signature in the TARGET dataset. The median riskscore was used as the cutoff value, and patients were split into high-risk (red) and low-risk (blue) groups. **(E)** Patients in the high-risk subgroup exhibited poorer progression-free survival compared to those in the low-risk subgroup.

Given the largest effective sample size along with detailed and standardized clinical characteristics in the TARGET-wt cohort, we chose this dataset as a discovery cohort to identify an immune signature. Univariate analysis using the Log-rank test showed 12 immune-related DEGs with prognostic ability (**Figure 3A**). Next, we used LASSO Cox regression analysis with 10-fold cross-validation to determine the optimal values of the penalty parameter and establish the most optimal prognostic signature. The ultimate resulted in eight nonzero coefficients when a coefficient profile plot was constructed against the log λ sequence (**Figures 3B, C**). An immune-related eight-gene model that reached an optimal prediction efficiency was ultimately obtained. Subsequently, by using independent regression coefficients of each gene, an eight-gene immune signature was established (**Supplementary Figure 2A**), and the riskscore was calculated as (0.4837)*GDF3 + (0.4567)*AVPR1B + (0.0342)*NR2F2 + (0.1737)*NODAL + (0.3124)*NRP2 + (0.4164)*OXT + (-0.2093)*FAM3B + (-0.5242)*EGF. The correlations of these constructed genes are shown in **Supplementary Figure 2B**. Additionally, we selected three public gene-expression datasets to verify the expression patterns of the eight genes (**Supplementary Figure 2C**). The riskscore of each patient was then computed using the aforementioned riskscore formula, and the patients were separated into a high- or low-risk subgroup based on their riskscore. Gene expressions in the signature lists of each patient were visualized using a heatmap (**Figure 3D**). In the TARGET cohort, WT patients in the high-risk subgroup had a statistically worse survival rate than those in the low-risk subgroup (**Figure 3E**). The time-dependent ROC analyses were applied to assess the predictive performance of this immune-related gene signature, and the values of area under the ROC curve (AUC) predicting 3-, 5-, and 7-year survivals were 0.83, 0.82, and 0.88, respectively (**Supplementary Figure 3A**).

### Association with clinicopathologic factors and construction of the nomogram and its accuracy verification

**Figure 4:**
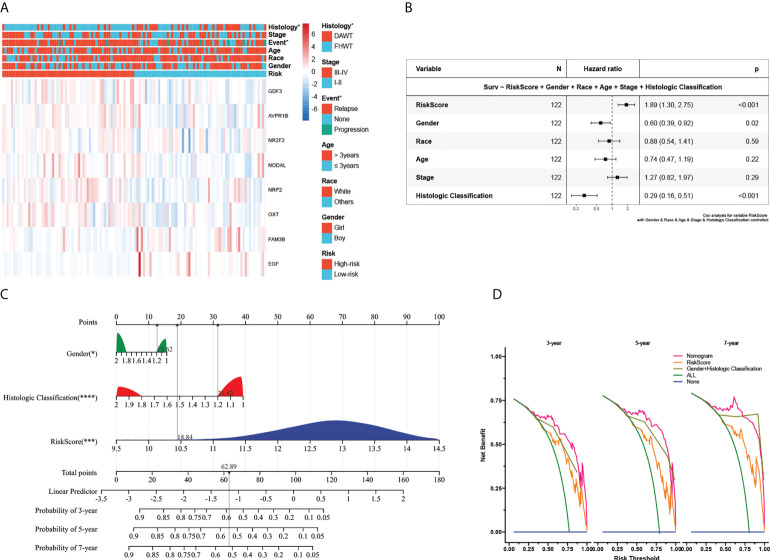
Association with clinicopathologic factors and construction of the nomogram and its accuracy verification. **(A)** Heatmap of the clinical relevance between the high- and low-risk subgroups in Wilms tumour. **(B)** The multivariate Cox regression analysis of risk factors in Wilms tumour. **(C)** The riskscore assessment nomogram to evaluate prognosis in Wilms tumour (3-, 5-, and 7-year survival rates). **(D)** On the x-axis, the calculated net benefit (y-axis) is displayed against the threshold probabilities of patients having 3-, 5-, and 7-year survival. The green line denotes the assumption that all patients have provided a survival time estimate. *, ***, and **** indicate a significance level of 0.05, 0.001, and 0.0001, respectively.

To further verify the signature’s clinical significance in WT patients, the relation between the immune signature and clinical and pathological characteristics was investigated. Here, we plotted a composite heat map to display the correlations of signature riskscore and clinicopathologic features. CDF3, AVPR18, NR2F2, NODAL, NRP2, and OXT were highly expressed in the high-risk subgroup, whereas FAM3B and EGF were highly expressed in the low-risk subgroup. The differences in pathological types and events between the high- and low-risk subgroups in the TARGET-wt cohort were likewise statistically significant (**Figure 4A**). Although we comprehensively examined publicly available data, regrettably, missing follow-up information resulted in the inability to validate the prognostic ability in an external dataset. Despite this, we still identified a cohort of 224 WT patients with favorable histology (FH) from the GSE31403 cohort (it contained data on recurrence but missed follow-up information). Subsequently, we calculated each patient’s riskscore from the GSE cohort using the developed risk model and then plotted the riskscore distribution. The patients were divided into two groups based on their median riskscore: high- and low-risk subgroups (**Supplementary Figure 4A**). Further analysis found that the high-risk subgroup was related to a higher clinical stage (P< 0.05) and tended to have a higher recurrent rate (31.25% vs. 23.21%). However, no statistically significant difference was observed in prognosis (P > 0.05), which was potentially relevant for the missing time variable (**Supplementary Figures 4B, C**).

Next, we explored its value for predicting clinical outcomes in WT patients. In univariate Cox proportional hazards analysis in the TARGET cohort, the riskscore of this signature was significantly associated with patients’ RFS (HR=2.72, 95%CI: 1.90-3.89, P< 0.001, **Supplementary Figure 4D**). In multivariate Cox regression analysis, after adjusting for the conventional clinical prognostic indicators (sex, age, race, clinical stage, and pathological type), the immune signature remained independently significant as a predictor of patients’ outcomes, suggesting that our model was stable and unaffected by clinical characteristics (**Figure 4B**). To explore the effect of the immune signature on prognosis in different subgroups with WT, we performed subgroup survival analysis. This signature’s resolving capacity for prognosis remained consistently stable in different subgroups classified by age, gender, clinical stage, and pathological type (**Supplementary Figure 5**).

For the prognostic capability of clinical indicators, the prognosis analysis from the TARGET cohort indicated that only sex and pathological type served as independent prognostic indicators for WT patients (**Figure 4B**). Notably, the prediction accuracy could be further improved using the full model that included both the signature riskscore and clinical prognostic factors. The AUC values under the ROC curves predicting 3-year survival were 0.83, 0.85, and 0.91, respectively (**Supplementary Figures 3A–C**). Together with the risk model and clinical features above, we constructed a nomogram to expand availability for clinical applications (**Figure 4C**, the constructed model passes the PH hypothesis test, detailed in **Supplementary File 5**). We assigned a riskscore to each patient by adding the points for each risk factor present, and a higher total score corresponds to a poor survival outcome. Moreover, the DCA showed that the nomogram has favorable clinical utilization (the C-index of the nomogram for RFS was 0.712), and a more net benefit was gained from the combined nomogram model compared with the signature alone or clinical model alone (**Figure 4D**).

### Associations between the immune signature and immune infiltration

**Figure 5:**
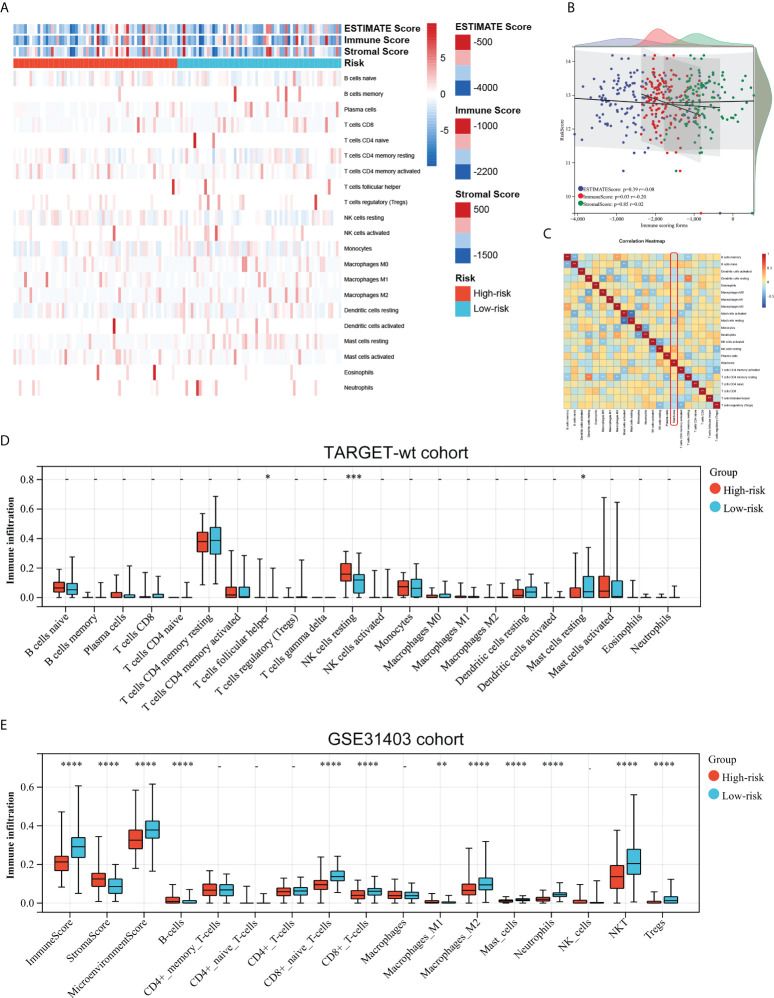
Associations between the immune signature and immune infiltration. **(A)** Landscape of the immune characteristics and tumour microenvironment in the TARGET-wt cohort. **(B)** Scatter plots depicting correlation of the immune-based risk signature with Stromalscore, Immunescore, and Estimatescore. **(C)** The correlation between 21 types of immune cells and riskscore in Wims tumour. **(D, E)** Distribution of immune-infiltrating cells in high- and low-risk subgroups in the TARGET-wt cohort and GSE31403 cohort. *, **, ***, and **** indicate a significance level of 0.05, 0.01, 0.001, and 0.0001, respectively.

As the prognostic signature was derived from the immune-related gene database (ImmPort), we hypothesized the immune signature could reflect the landscape of immune infiltration. Thus, a heatmap was generated to visualize immune cell subpopulations’ immune score and relative abundances in the TARGET dataset by ssGSEA (**Figure 5A**). The ESTIMATE analyses showed that the riskscore was negatively correlated with the Immunescore (**Figure 5B**). **Figure 5C** showed the correlation between the infiltration abundances of 22 immune cells and riskscore. Of greatest concern, compared with the low-risk subgroup, the high-risk subgroup had a lower level of T cells follicular helper, and Mast cells resting, and a higher level of natural killer (NK) cell resting (**Figure 5D**). This means high-risk patients were in an immunosuppressed tumour microenvironment. To explain the survival differences found in patients with favorable outcomes from a perspective of tumour immune, we next further analyzed the difference of immune cell infiltration between high- and low-risk patients with FH-WT in the GSE31403 cohort. The Immunescore, microenvironment score, and main lymphocyte subsets involved in anti-tumour immunity, particularly CD8^+^T cell, Macrophages_M2, Master cells, Neutrophils, NKT cell, and Tregs were significantly decreased in the high-risk subgroup (**Figure 5E**). These results suggest that a favorable prognosis may in part be attributed to the activity of anti-tumour immunity.

### Mutational landscape of the signature and its effect on the immune response

**Figure 6:**
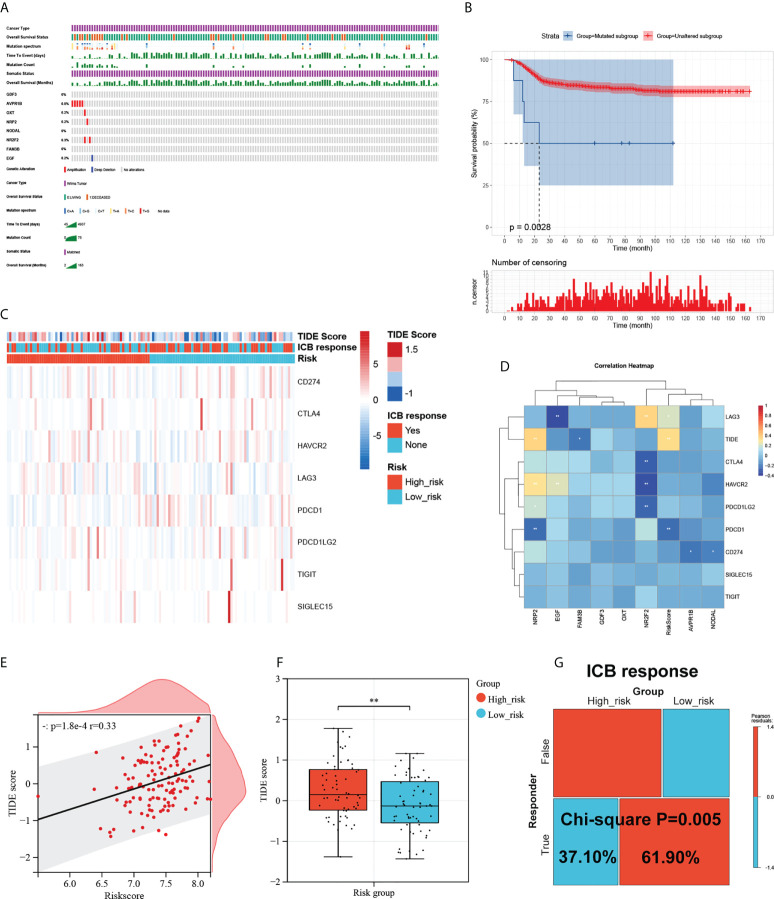
Mutational landscape of the signature and its effect on the immune response. **(A)** Mutational landscape of the immune-genes in TARGET-wt cohort. **(B)** Survival analysis of the mutated subgroup versus unaltered subgroup in TARGET-wt cohort was provided using Kaplan-Meier curve. **(C)** Heatmap of immune checkpoint molecules’ expression, TIDE score, and ICB response based on the signature. **(D)** The correlation heatmap of eight immune-genes and riskcore with immune checkpoint molecules and TIDE score. **(E)** Scatter plots depicting correlation of the immune-based risk signature with TIDE score. **(F)** Box plot showing the differences of TIDE score between the high- and low-risk subgroups in TARGET-wt cohort. **(G)** Immune response difference between the high- and low-risk subgroups based on TIDE score in TARGET-wt cohort. *, and ** indicate a significance level of 0.05 and 0.01, respectively.

To investigate the mutational landscape of the gene signature in WT, genetic alterations were analyzed using cBioPortal (**Figure 6A**). Survival analysis indicated a greatly decreased RFS in the mutated subgroup compared to the wild-type subgroup (**Figure 6B**). Tumour cells are able to evade immune surveillance and develop through a variety of mechanisms, including the overexpression of inhibitory immune checkpoint molecules, which inhibit anti-tumour immunological responses (39). ICB therapy has emerged as a revolutionary immune-based cancer therapy. Here, the TIDE score and the expression patterns of immune checkpoint molecules were displayed using a Heatmap visualization method (**Figure 6C**). Results showed a correlation between riskcsore, multiple risk genes, and immune checkpoint molecules and TIDE score (**Figure 6D**). Next, based on simulations of tumour immune escape mechanism, we used the TIDE algorithm to predict the response to immunotherapy in TARGET cohort. Surprisingly, the results show a close positive correlation between TIDE score and riskscore (**Figure 6E**). Further analyses exhibited that the TIDE score of patients in the low-risk subgroup was significantly lower than those in the high-risk subgroup (**Figure 6F**), showing a higher response rate to ICB treatment (**Figure 6G**). These results provide further evidence that patients in the low-risk subgroup have more favorable prognoses than high-risk patients and hold a greater potential for immunotherapy applications.

### Biological function related to the immune signature and small molecule drugs exploration

**Figure 7:**
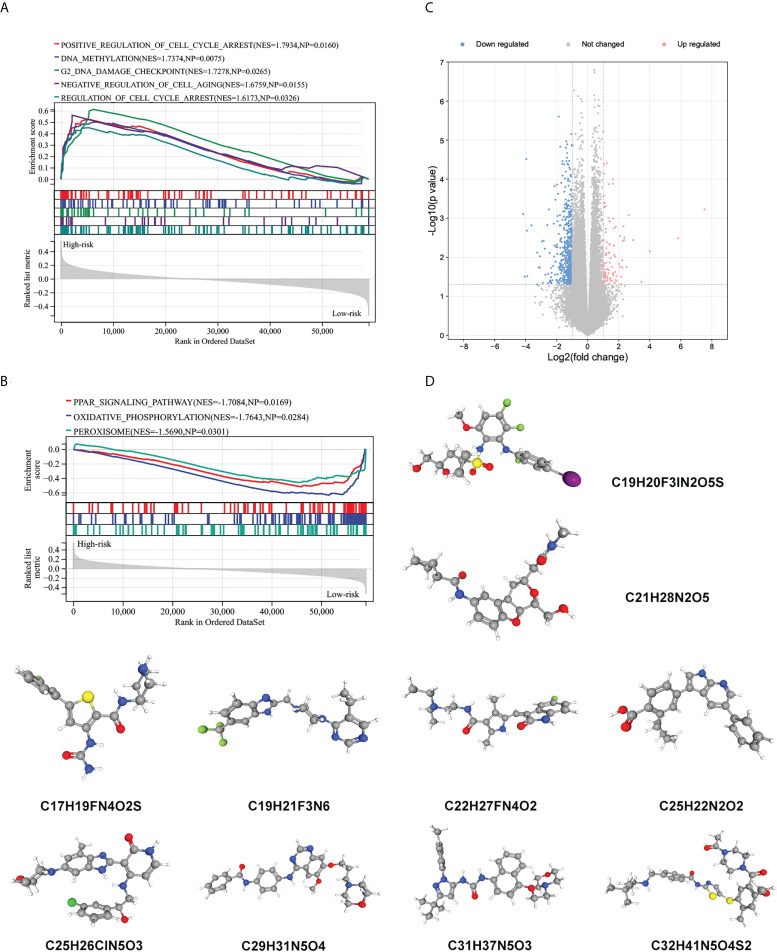
Biological function related to the immune signature and small molecule drugs exploration. **(A, B)** Gene set enrichment analysis of significantly enriched pathways and biological functions in the high-risk subgroup **(A)** and low-risk subgroup **(B)**. **(C)** Genes that are expressed differently in high- and low-risk subgroups. **(D)** The 3D structure of prospective drugs selected from the cMap and DeSigN database.

To further explore potential biological processes and pathways enriched by the immune signature, we performed grouped GSEA with TARGET datasets. GSEA results show significant enrichment of cell cycle, DNA methylation, cell cycle checkpoint, and cell aging pathways in the high-risk subgroup, and PPAR signaling pathway, oxidative phosphorylation, and peroxisome pathways in the low-risk subgroup (**Figures 7A, B**). Correlation analysis showed that several risk genes including AVPR1B, NR2F2, NODAL, NRP2, OXT, FAM3B, and EGF were associated with the expression of several DNA damage response-related genes (**Supplementary Figure 6**). Moreover, small molecule drugs were explored for WT using the CMap and DeSigN database. There were 101 genes that were up-regulated and 538 genes that were down-regulated when the low- and high-risk subgroups were compared (**Figure 7C**). Based on the differentially expressed genes, the two most relevant drugs were investigated as prospective candidates for WT patients (**Figure 7D**).

### The expression of NRP2, EGF, NODAL, and NR2F2 in different Wilms tumour cell lines

**Figure 8:**
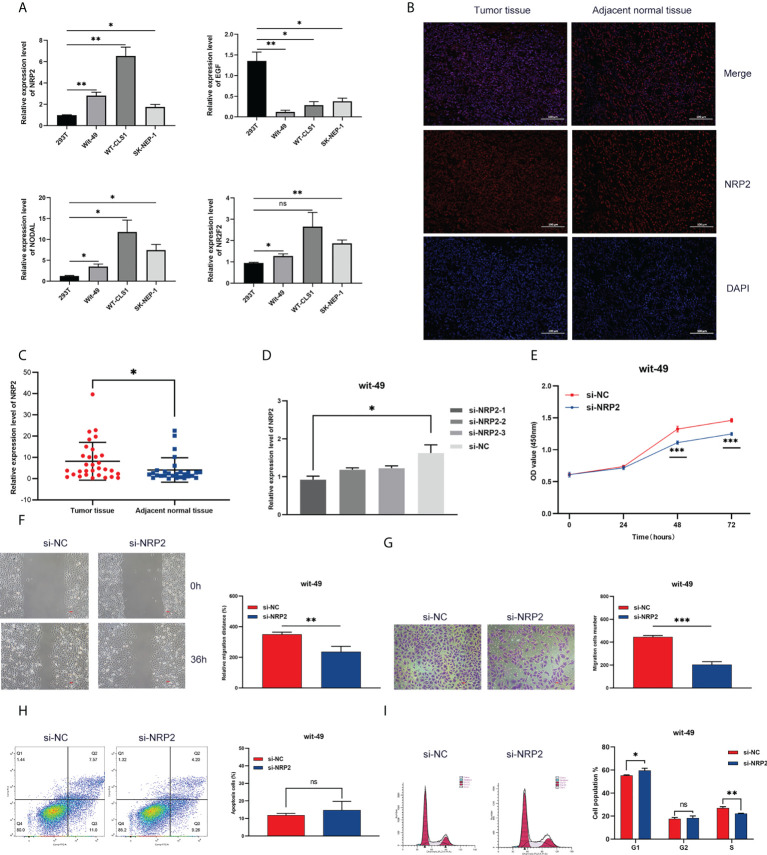
Expression and functional validation. **(A)** The mRNA expression of NRP2, EGF, NODAL, and NR2F2 in different Wilms tumour cell lines. **(B)** The difference in NRP2 expression patterns was confirmed by immunofluorescence staining in tumour tissue and non-tumour tissue. **(C)** The expression level of NRP2 in tumour tissue and non-tumour tissue. Results were analyzed by unpaired t-test. **(D)** qRT-PCR analysis of NRP2 expression in wit-49 cells after transfection with siRNA. **(E-G)** The proliferation, migration, and invasion of wit-49 cells by the CCK-8 assays, wound healing assays, and transwell assays. **(H, I)** Cell cycle phase distribution and cell apoptosis rate were determined by flow cytometry. *, **, and *** indicate a significance level of 0.05, 0.01, 0.001, respectively; and ns indicate no significant level.

We screened four risk genes with high expression abundance from the immune signature for verification by qRT-PCR using different tumour cell lines (Wit-49, WT-CLS1, and SK-NEP-1) and a normal renal epithelial cell line (293T). According to the RNA-seq analysis, NRP2, NODAL, and NR2F2 were highly expressed, while EGF was lowly expressed in tumour tissue. The result of qRT-PCR assays validated these trends (**Figure 8A**).

### Silencing of NRP2 suppressed proliferation, invasion, and migration in WT cells

Among these genes, NRP2 plays a critical regulatory role in the progression, drug resistance, and immune response of a variety of tumours, as presented in the discussion. NRP2 was then selected for further functional validation in WT cells. NRP2 is abundantly expressed in WT tissues relative to nearby non-tumour tissues, as previously observed (**Figures 8B, C**). We created three siRNA sequences for NRP2 silencing to explore its functional involvement in WT carcinogenesis, and the one with the best inhibitory effect (si-NRP2-1) was used (**Figure 8D**). In wit-49 cells, si-NRP2-1 significantly decreased NRP2 levels when compared to the negative control siRNA. The CCK-8 assay revealed that silencing NRP2 greatly decreased the capacity of WT cells to proliferate (**Figure 8E**). Furthermore, after NRP2 was silenced, cells demonstrated a reduction in their capacity to migrate and invade (**Figures 8F, G**). Cell cycle distribution analysis revealed that the fraction of cells in S-phase reduced dramatically while the proportion in G1-phase was increased (**Figure 8I**). However, silencing of NRP2 did not affect the proportion of apoptotic cells (**Figure 8H**). These results suggest the potential role of NRP2 in WT carcinogenesis.

## Discussion

Wilms tumour is the most prevalent primary kidney malignancy of childhood, which is becoming a multi-modal treatment paradigm for pediatric solid tumours. The stepwise advances in treatment by the National Wilms tumour Study Group (NWTSG), which has been succeeded by the Children Oncology Group (COG), and the International Society of Pediatric Oncology (SIOP) have improved the survival rate to 90% today (2–4). Initial studies from NWTSG and SIOP relied on clinical risk factors to determine the treatment protocols. Over time, more risk factors could be incorporated into risk stratification criteria to achieve risk-oriented individualized treatment of patients (40–44). However, these methods are applicable to a specific subgroup and not sensitive enough for clinical application. With the rapid development of bioinformatics in recent years, numerous prospective biomarkers either for diagnosing or prognosis predicting were identified *via* wide-scale transcriptome RNA sequencing data and clinical resources. A more effective and accurate assessment facilitates individualized treatment in both research and clinical practice. Although previous studies have identified numerous single-gene biomarkers associated with prognosis, few of them have been applied in a clinical setting. Given cancer biology’s complex and interactive processes, a polygenic signature could provide better prognostic reliability than a unigene feature. Also, the role of the tumour microenvironment in carcinogenesis and progression has been highlighted in recent studies (45, 46). Immunotherapies, including those that target the immune checkpoint, have demonstrated positive results in various malignancies (47). Recently, a follow-up study among an independent cohort found PD-L1 overexpression was associated with poorer OS and DFS in WT patients (48). Consequently, we envisage that immunotherapy may be the most effective means to promote survival rates and quality of life for WT patients. Nevertheless, there are no prognostic markers for evaluating the immunologic status of the TME and predicting responses to immunotherapy. Thus, as the original aim of our study, we proposed to develop a series of immune-related biomarkers. In this study, we successfully establish an immune-related gene signature reflecting immunophenotypic heterogeneity to predict outcomes and responses to immunotherapy in WT patients.

We started the present study with differentially-expressed genes associated with immunity. It is necessary to define immune-related biomarkers from the perspective of tumour immunity, which could help select potential candidates for immunotherapy. We submitted these genes to a Lasso penalized Cox regression analysis for establishing an eight immune-related gene signature and further established a risk scoring system. We then demonstrated the significant differential expression of the eight genes between tumour and normal tissue, which generally agrees with public datasets. In addition, we assessed the expression of four IRGs among them in three human Wilms tumour cell lines and compared them with a normal renal epithelial cell line, suggesting potential targets for further study. Numerous independent studies have shown the expression level of several single-gene was strongly correlated with the prognosis of WT patients (49–52). Additional information of comprehensive bioinformatics analysis, clinicopathological factors, and model analysis were needed to further enhance the prediction accuracy of the prognosis. Recently, gene signatures based on aberrant mRNA attracted wide attention as potential biomarkers of the prognosis of cancer patients (53). Here, all patients were separated into a high- and low-risk subgroup using this eight-gene prognostic signature. The model showed a promising value in predicting both RFS risk and clinicopathological features. Univariate and multivariate Cox regression analyses indicated that the signature was an independent prognostic factor for WT patients, supporting the signature as a reliable predictive tool. More than that, we testify the correlation between the signature and clinicopathological features on the largest publicly chip dataset from GEO. Additionally, gender and pathology type were the independent prognostic factors affecting RFS in our study as well. To offer clinicians a quantitative approach to predicting the prognosis of WT patients, we integrated clinical characteristics with the signature to construct a combined nomogram model, which could more accurately predict their short-term and long-term survival.

Recent literature highlights the TIM as a complex milieu in which imbalances between tumours and the host immune response can lead to tumour progression (46). Understanding the immunological state of the TME will facilitate us to deepen our understanding of the anti-tumour immune response and develop more effective immunotherapy approaches. Tumour-infiltrating immune cells are a critical part of the TME. Accumulating evidence has issued its clinical and pathological significance in predicting prognosis and therapeutic response (54). Immune-related gene signature or other types of signature was a new tool for the exhaustive evaluation of TME and effective biomarkers to predict prognosis and guide immunotherapy (55–57). We confirmed that the high-risk subgroup was significantly associated with characteristics related to the TME, especially immune infiltration. By evaluating the level of immune cells infiltration in the TME, we noticed two primary features: 1) executive lymphocytes of anti-tumour (such as CD4^+^ T cells, CD8^+^ T cells, NK cells, and NKT cells) responses presented immunosuppressive status which feedback-induced M1 to M2 macrophage polarization; 2) Pearson correlation coefficient analysis showed that the riskscore is negatively correlated with the Immunescore. Hence, impaired anti-tumour immunity in high-risk patients might be the reason for their unfavorable prognosis. Insights into the mechanisms that sabotage anti-tumour immune responses will aid the development of more effective therapeutics. In short, the novel immune-related risk signature may represent the immune status of the WT patient and could serve as a biomarker to predict immunotherapy efficacy.

In adult oncology, the study of the TIM has exhibited great potential in revealing new prognostic biomarkers as well as new therapeutic options and represents a significant advantage with the benefit of reduced toxicity over traditional chemotherapy. The introduction of immunotherapy into the field of pediatric oncology has been met with enthusiastic efforts, although with some delay. Immunotherapy is expected to become a promising choice for 10% of patients that were resistant to currently available therapies. On the other hand, in pediatric oncology, the early and late toxicities of cytotoxic chemoradiotherapy lead to severe complications until adolescence and adulthood (8). Promisingly, immunotherapy provides a unique opportunity to create novel treatment strategies that could be rapidly implemented in the clinic. Due to the inhomogeneity and interaction of anti-tumour immune responses and cancer biology, one single biomarker was unable to sufficiently predict response to immunotherapy (58, 59). A lack of greater understanding of tumour immunology may be an underlying cause of failure for most immunotherapies to date. Therefore, we need a novel prognostic biomarker to improve the current risk stratification system to identify patients who are more likely to benefit from immunotherapy. In addition, the regulation and expression of immune checkpoint molecules (such as PD-1 and PD-L1) also serve a critical surveillance role in regulating immune responses by inhibiting the activation of protective immune cells and promoting immune responses (60). High expression of immune checkpoint molecules frequently benefits more from immune checkpoint inhibitor therapy. Our results revealed a significant correlation between riskscore and the crucial immune checkpoints PDCD1 (also known as PD-1) and LAG3. Cumulative studies have shown them to be important targets for immunotherapy (61). More importantly, this study is built upon the immune-related genes; we theorized there might be a possible association between the immune signature and the efficacy of immunotherapy. Combined with TIDE algorithm analysis, we explore the correlation between riskscore and ICB immunotherapy response in the TARGET-wt cohort. TIDE results presented that riskscore positively correlated with TIDE score, and more immunotherapeutic responders appeared in the low-risk subgroup than in the high-risk subgroup, which means low-risk WT patients with a lower TIDE score are more promising in responding to ICB. These findings further reveal that the signature based on immune-related genes can help predict patients’ outcomes and identify optimal candidates for immunotherapy.

By searching in PubMed, we found important roles of these genes in tumours. Several studies have shown that high expression of FAM3B promoted progression in the colon, prostate, and esophageal cancer and drug resistance in gastric cancer (62–65). A therapeutic cancer vaccine (CIMAvax-EGF) developed based on EGF in lung cancer patients showed good tolerability and survival benefit in clinical trials (66, 67). NR2F2 played a key role in the proliferation and invasion of gastric and breast cancer cells through its effect on MET (68, 69). NODAL is an embryonic protein involved in TGF-β signaling and is highly expressed in various cancers. Cancer cells with high levels of NODAL displayed a more aggressive phenotype *in vitro*, while *in vivo* was associated with a poorer prognosis in several human cancers, highlighting it as a potential target and marker for targeted cancer therapy (70–72). Previous studies have also identified a novel link between OXT and cancer. OXT inhibited ovarian cancer metastasis by suppressing the expression of MMP-2 and VEGF (73). Interestingly, stimulation of hypothalamic OXT neurons inhibited the progression of colorectal cancer in mice (74). Thus OXT may be a new strategy for the treatment of colorectal cancer. Moreover, GDF3 played a paradoxical role in cancer that it inhibited the growth of breast cancer cells and promotes paclitaxel-induced apoptosis (75); on the other hand, it promoted the progression of melanoma (76).

Among these genes, NRP2 has been most intensively studied in tumours. As a non-tyrosine kinase receptor, NRP2 is frequently overexpressed in various malignancies, and associated with many pro-cancer behaviors. In addition, NRP2 has been found to be involved in tumour development through a variety of novel pathways. For example, in prostate cancer, this gene regulated osteoclast differentiation and function, thus, targeting this gene could be beneficial in the treatment of prostate cancer bone metastases (77). NRP2 also acted as a regulatory target for a variety of non-coding RNA to enhance the malignant behavior of therapeutic cells (78–84). In TIM, cancer-associated fibroblasts (CAFs) promoted gastric cancer chemoresistance through NRP2 expression (85); NRP2 in macrophages promoted tumour growth by regulating the cytostatic effects of apoptotic tumour cells and coordinating immunosuppression (86). Mesenchymal-epithelial transition (MET) is known to be an essential process involved in tumour cell invasion and metastasis, and NRP2 contributes significantly to TGF-β1-induced EMT in lung cancer (87). Emerging studies suggested that the NRP2/WDFY1 axis was required to maintain endocytic activity in cancer cells, and therefore, therapeutic targeting of endocytosis may be an attractive strategy to selectively target cancer cells in a variety of malignancies (88). Furthermore, a major study has shown that blocking NRP2 function selectively disrupted the vascular endothelial growth factor (VEGF)-induced lymphatic endothelial cell migration, but not proliferation, which did not affect established lymphatic vessels in mice but decreased tumour lymphatic vessel generation, and metastasis of anterior lymph nodes. These results suggest that NRP2 could be an attractive target for regulating metastasis (89). In this study, silencing of NRP2 inhibited proliferation, invasion, and migration in WT cells, suggesting that NRP2 had an oncogenic impact in WT cells. These findings offer strong evidence of NRP2’s critical function in human malignancies.

We believe that the established prognosis signature is inherently immunological, and stratification of patients based on immunophenotype might prove useful. We, therefore, explored the downstream mechanism involved in the different immunophenotypes and showed that the phenotypic regulation of the cell-fate decisions in high- and low-risk subgroups was potentially regulated *via* affecting DNA methylation, cell cycle, and cellular metabolism. The relevant potential drugs were also predicted based on the significantly differentially expressed genes between the two risk groups. Of these, Refametinib (C19H20F3IN2O5S) and Sunitinib (C22H27FN4O2) have tremendous therapeutic potential in a variety of adult tumors (90–94). However, children are not ‘small adults’. Pediatric tumours are likely to follow a unique immuno-oncologic mechanism. Further investigation of these issues will help in understanding molecular mechanisms leading to immune evasion in WT and provide a rational basis for novel therapies in the future. These areas remain to be explored.

To our knowledge, this is the first study involving the prognostic signature and immune characteristics. The present results can contribute to improving the clinical risk evaluation of WT patients and offer new perspectives for future research on neoadjuvant therapy. However, some limitations ought to be considered in generalizing the present study’s findings. Firstly, this study is a retrospective review of public datasets; selection bias is inherent to the design. Thus, large and longitudinal prospective studies will be necessary to test this hypothesis before it can be implemented in clinical practice. Secondly, since immunotherapy has not been widely developed in WT, the patients’ response to immunotherapy was predicted by TIDE analysis. Finally, although functional analysis revealed several potential immune-related mechanisms, the exact mechanism remains to be explored. Overall, additional research should be developed to clarify these hypotheses that hold the promise of improving the prognosis of WT patients.

In summary, the present study identified a novel immune signature, which could predict the patients’ outcomes and characterize the immune status. The signature can help risk-adjusted personalized treatment and identify optimal candidates for immunotherapy. Potential drugs were also forecasted based on the differentially expressed genes. The drugs’ therapeutic efficacy and the underlying mechanisms linking signature and tumour immunity in WT remained unexplored and warranted further investigation.

## Data availability statement

The original contributions presented in the study are publicly available. This data can be found here: https://www.ncbi.nlm.nih.gov/geo/query/acc.cgi?acc=GSE197047.

## Ethics statement

The studies involving human participants were reviewed and approved by The Ethics Committee of Children’s Hospital of Chongqing Medical University. Written informed consent to participate in this study was provided by the participants’ legal guardian/next of kin.

## Author contributions

FL and G-HW contributed to conception and design. TL, FL, and G-HW contributed to administrative support. Q-LS, M-LC, CZ, and Z-XZ contributed to collection and processing of clinical specimens. X-MT, BX, and L-MJ contributed to experimental process; X-MT, TM, and J-KW contributed to bioinformatics analysis. X-MT, J-KW, Q-LS, TM, and BX contributed to preparing all the Figures and tables. X-MT and BX contributed to manuscript writing. All authors contributed to the article and approved the submitted version.

## Funding

This work was supported by the Natural Science Foundation of Chongqing (cstc2021jcyj-msxmX0345 and cstc2021jcyj-msxm-X0549), the Medical Scientific Research Project of Chongqing (NO.2022GDRC009), the general project of clinical medicine research of Children’s Hospital of Chongqing Medical University (NCRC-2019-GP-08).

## Conflict of interests

The authors declare that the research was conducted in the absence of any commercial or financial relationships that could be construed as a potential conflict of interest.

## Publisher’s note

All claims expressed in this article are solely those of the authors and do not necessarily represent those of their affiliated organizations, or those of the publisher, the editors and the reviewers. Any product that may be evaluated in this article, or claim that may be made by its manufacturer, is not guaranteed or endorsed by the publisher.
