# Supplementary figures and images for: Immune-related gene signature associates with immune landscape and predicts prognosis accurately in patients with Wilms tumour
Source: Front Immunol. 2022 Sep 12;13:920666. doi: 10.3389/fimmu.2022.920666 (PMC9510599; doi:10.3389/fimmu.2022.920666)

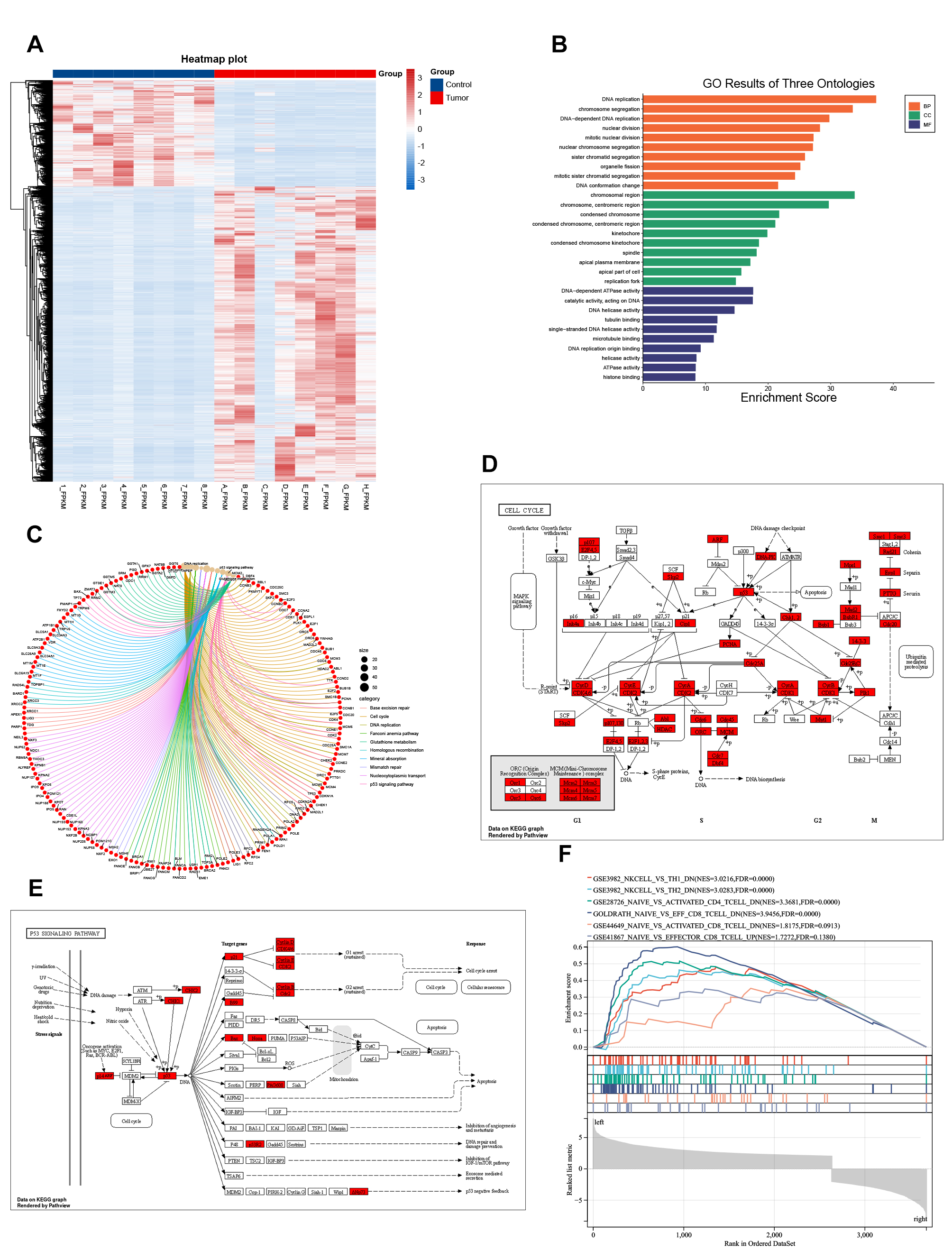

Supplement: Supplementary file 1 [file Image_1.jpeg]

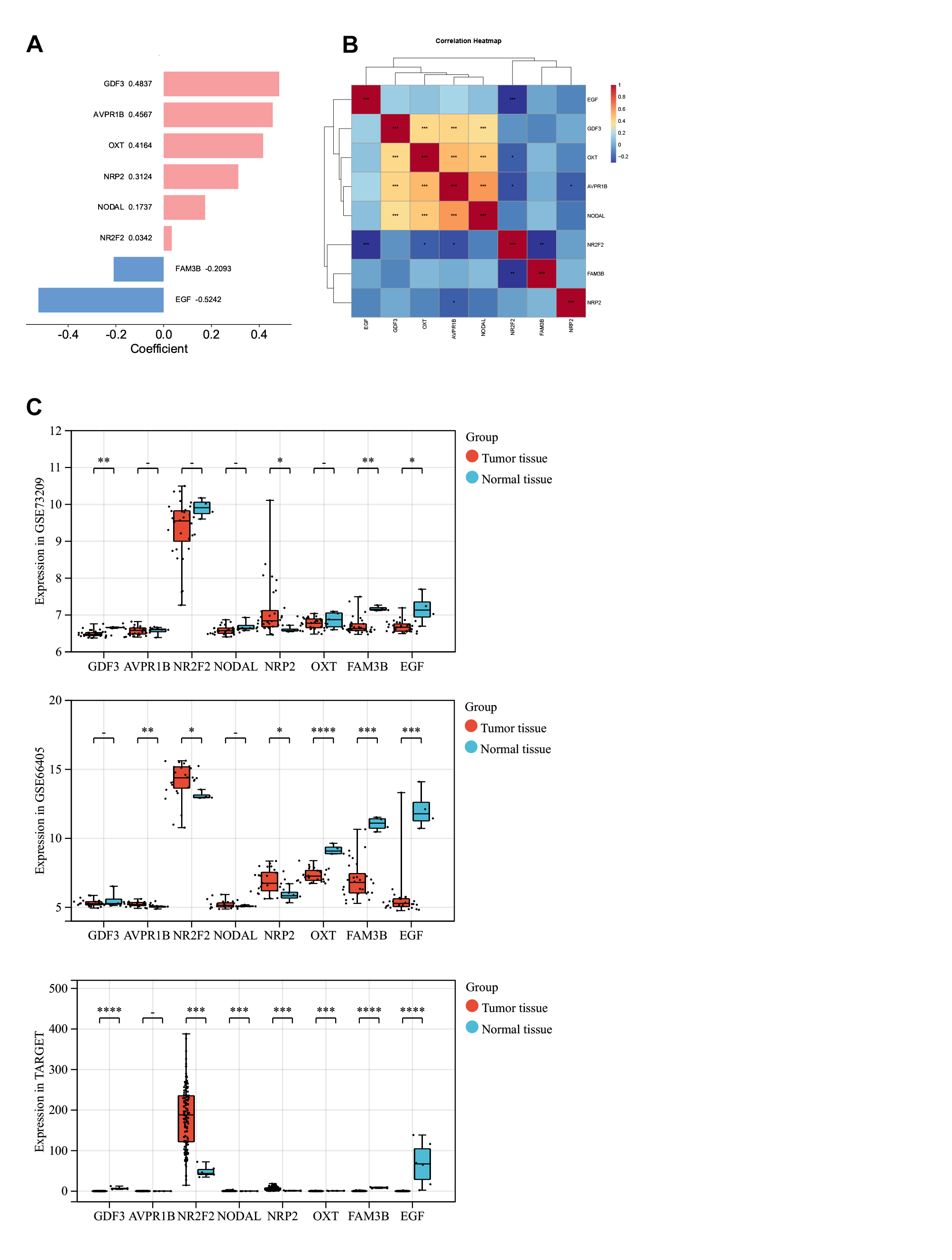

Supplement: Supplementary file 2 [file Image_2.jpeg]

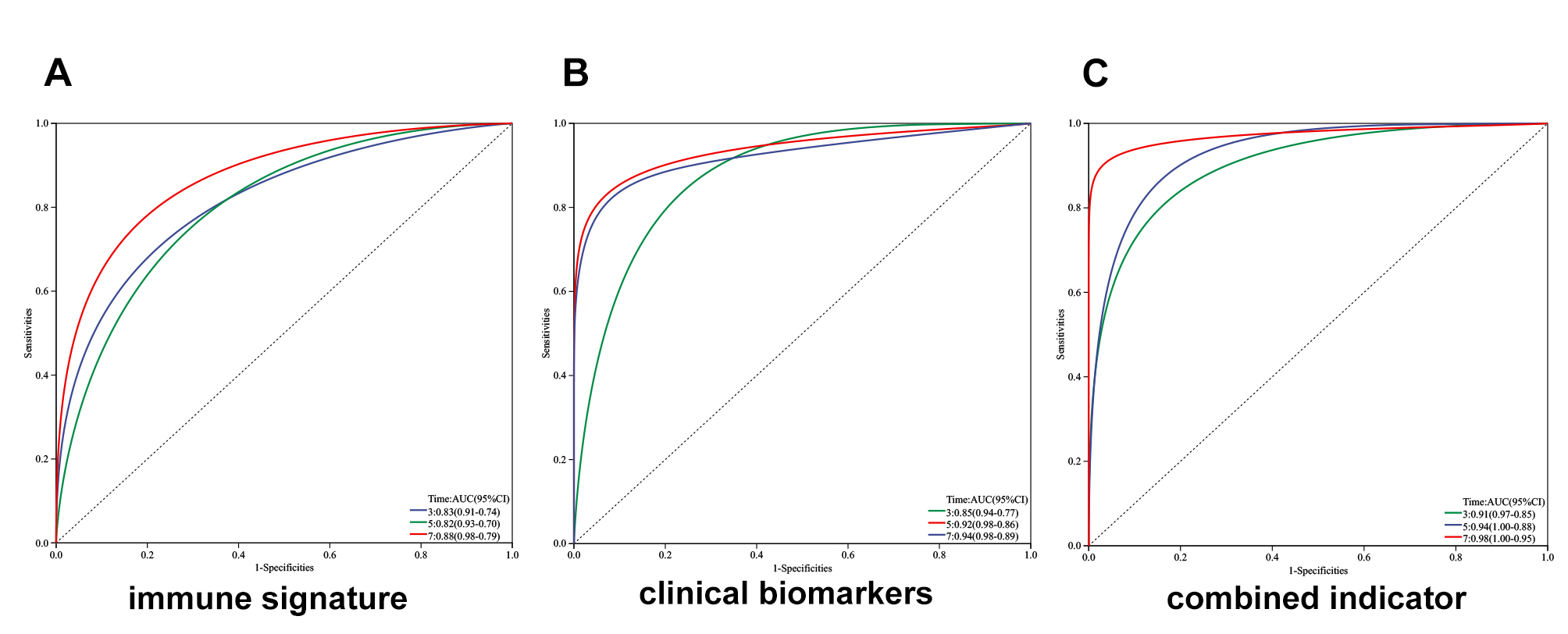

Supplement: Supplementary file 3 [file Image_3.jpeg]

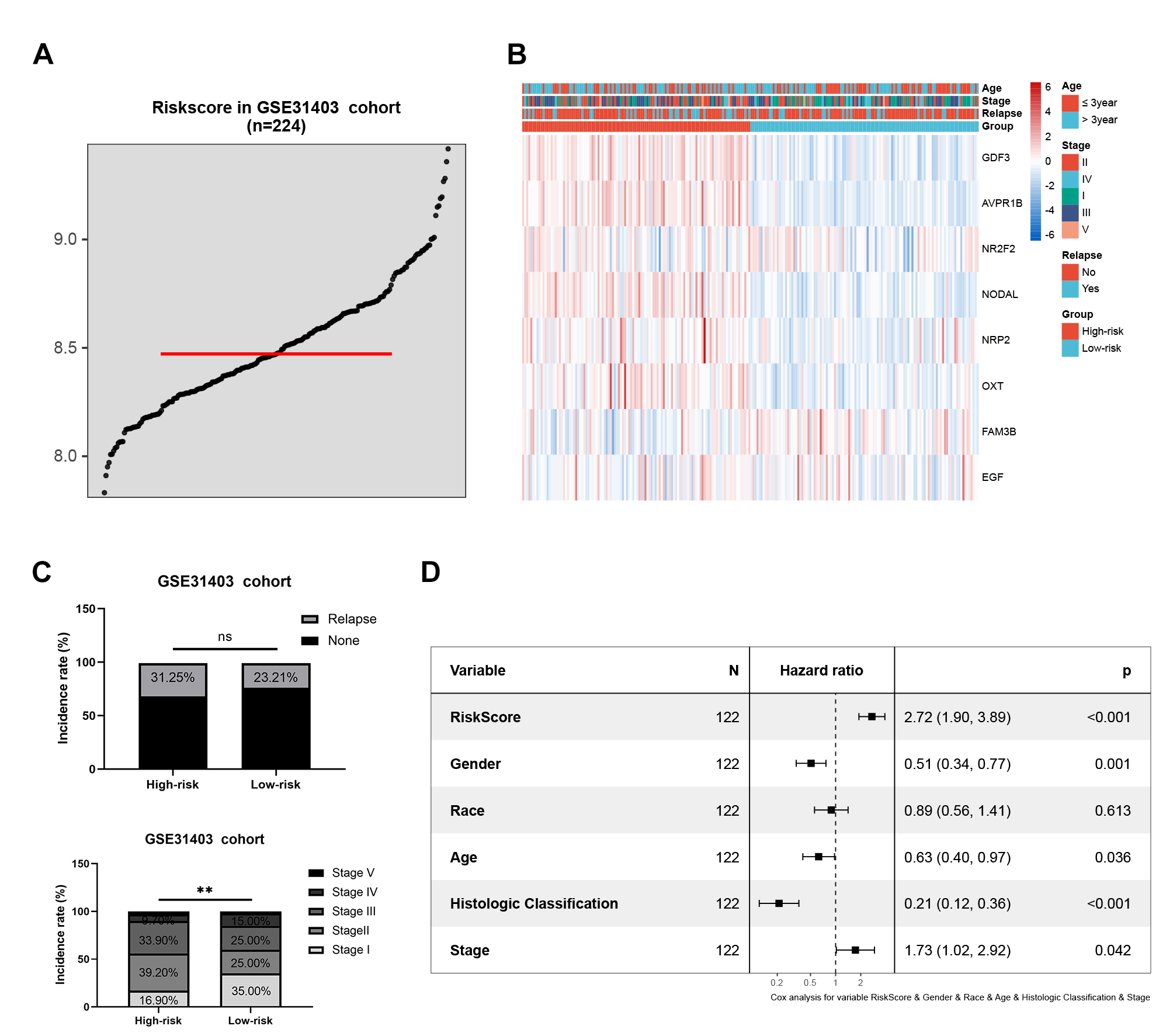

Supplement: Supplementary file 4 [file Image_4.jpeg]

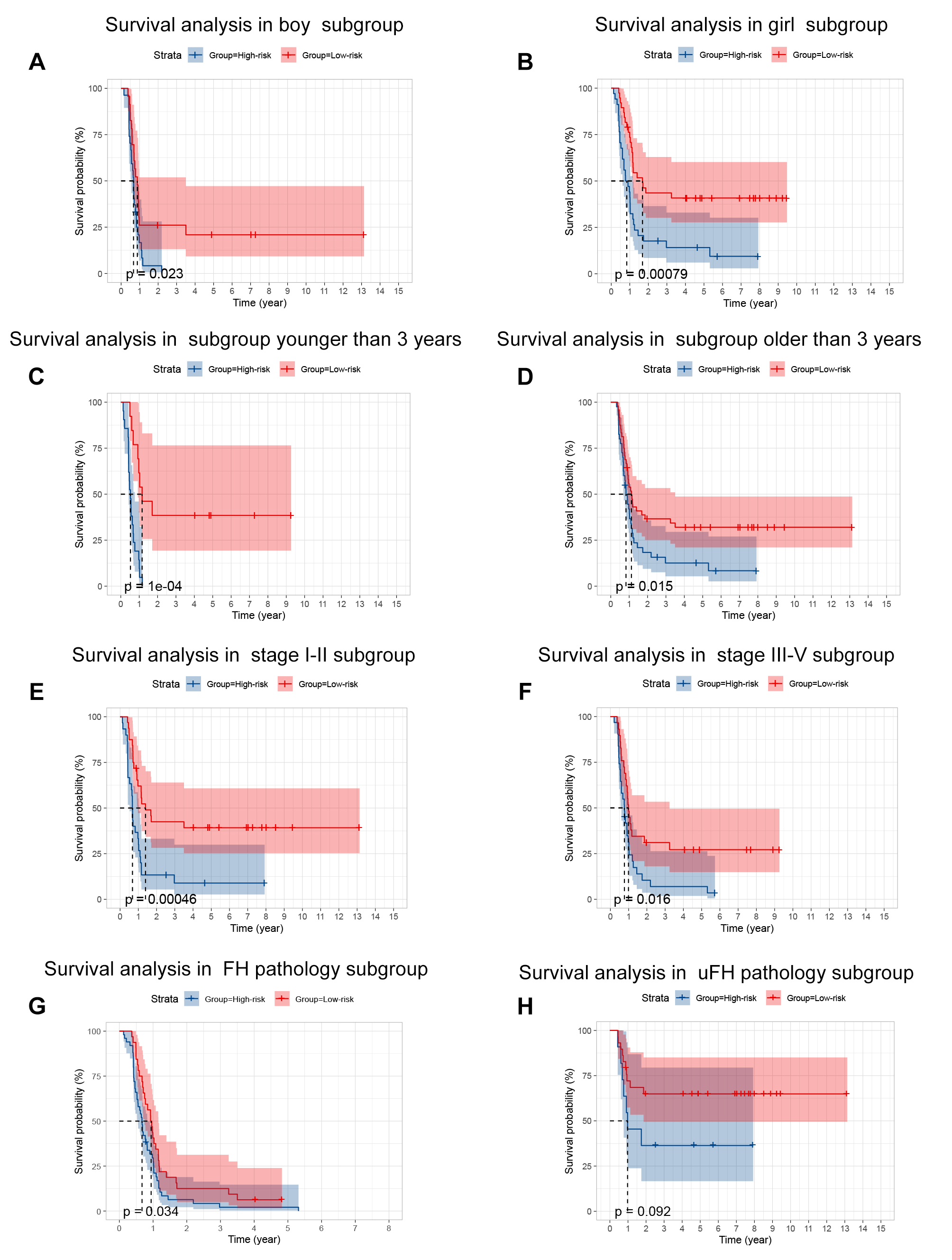

Supplement: Supplementary file 5 [file Image_5.jpeg]

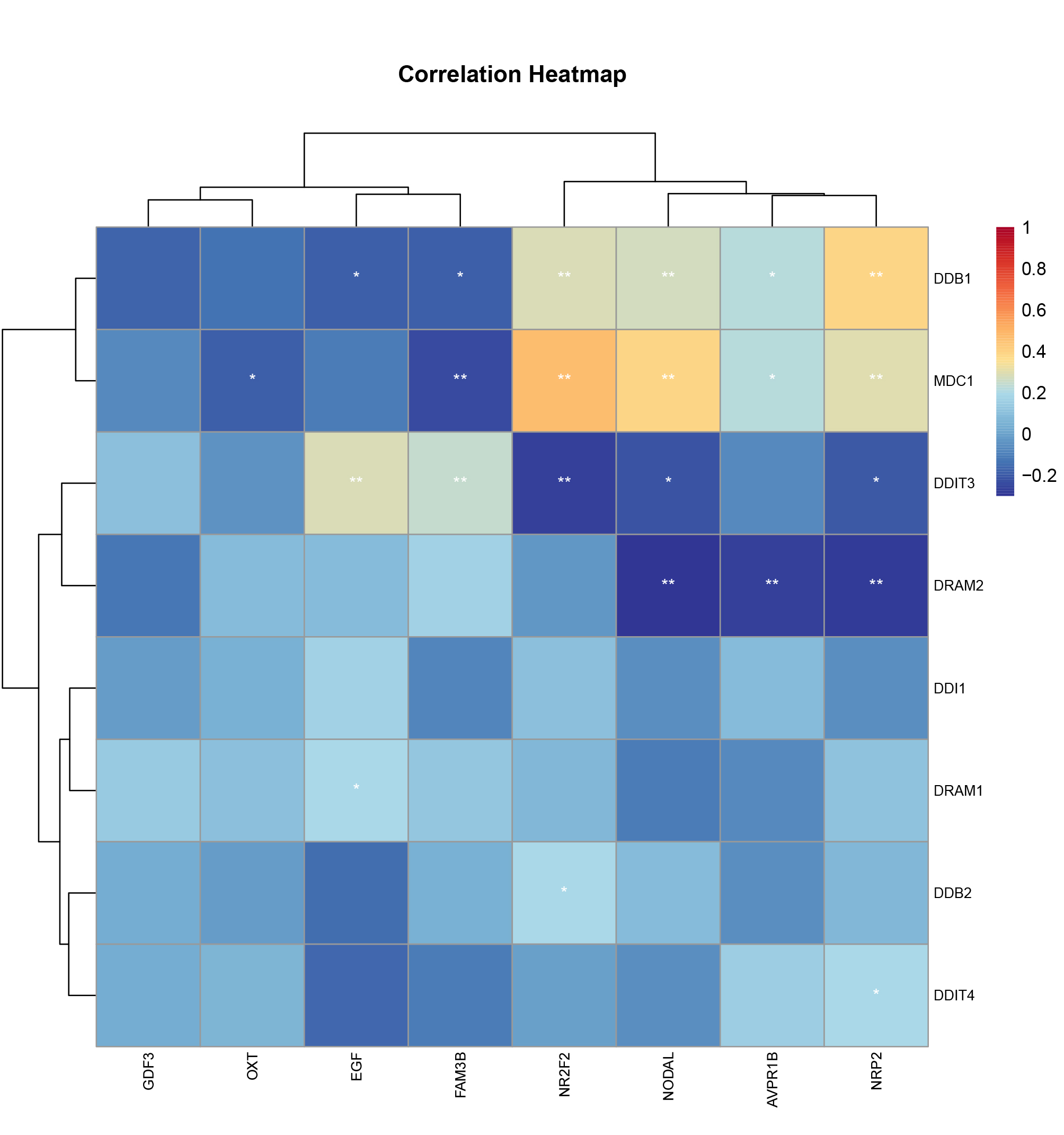

Supplement: Supplementary file 6 [file Image_6.jpeg]
