# Supplementary material for: Immune-related gene signature associates with immune landscape and predicts prognosis accurately in patients with Wilms tumour
Source: Front Immunol. 2022 Sep 12;13:920666. doi: 10.3389/fimmu.2022.920666 (PMC9510599; doi:10.3389/fimmu.2022.920666)
Supplement: Supplementary file 10 [file Table_4.docx]

Table 1. Primers used for quantitative real time PCR.

| RNA | Forward primer | Reverse primer |
| --- | --- | --- |
| NR2F2 | TCATGGGTATCGAGAACATTTGC | TTCAACACAAACAGCTCGCTC |
| NODAL | CTGCTTAGAGCGGTTTCAGATG | CGAGAGGTTGGAGTAGAGCATAA |
| NRP2 | ATCATCCTGCCCAGCTACGA | AAATTCTCACCTGCAAAAGCCG |
| EGF | TGGATGTGCTTGATAAGCGG | ACCATGTCCTTTCCAGTGTGT |
| GAPDH | GGAGTCCACTGGCGTCTTCA | GTCATGAGTCCTTCCACGATACC |
