# Supplementary material for: Immune-related gene signature associates with immune landscape and predicts prognosis accurately in patients with Wilms tumour
Source: Front Immunol. 2022 Sep 12;13:920666. doi: 10.3389/fimmu.2022.920666 (PMC9510599; doi:10.3389/fimmu.2022.920666)
Supplement: Supplementary file 11 [file Table_5.docx]

| PH hypothesis testing | | |
| --- | --- | --- |
| Variables | χ2 | *p* value |
| RiskScore | 0.137 | 0.711 |
| Histology-1.0[ref] | - | - |
| 2.0 | 3.753 | 0.053 |
| Gender-1.0[ref] | - | - |
| 2.0 | 0.045 | 0.833 |
